# Efficiency of Early Warning Scores in Adult Emergency Department and Inpatient Populations: Overview of Reviews

**DOI:** 10.5811/westjem.62022

**Published:** 2026-08-31

**Authors:** Michal Pospíšil, Tereza Friessová, Tamara Skříšovská, Andrea Pokorná, Jan Maláska, Jana Rozmarinová, Alena Langaufová

**Affiliations:** * Masaryk University, Faculty of Medicine, Department of Health Sciences, Brno, Czech Republic; † University Hospital Brno and Masaryk University, Faculty of Medicine, Department of Paediatric Anaesthesiology and Intensive Care Medicine, Brno, Czech Republic; ‡ Masaryk University, Faculty of Medicine, Department of Simulation Medicine, Masaryk University, Brno, Czech Republic; § University Hospital Brno, Second Department of Anaesthesiology and Intensive Care Medicine, Brno, Czech Republic

## Abstract

**Introduction:**

Early Warning Scores (EWS) are used to support decisions regarding triage and early intervention of patient deterioration in emergency departments (ED) and other clinical environments. We synthesized evidence from systematic reviews on the predictive performance and clinical impact of EWS and their modifications in adult in-hospital populations.

**Methods:**

We conducted and reported an overview of reviews following the Joanna Briggs Institute (JBI) methodology, PRIOR statement, and our predefined protocol. A systematic search of MEDLINE (Ovid) and Epistemonikos was performed in May 2025. Two reviewers independently screened studies, extracted data, and critically appraised systematic reviews. We included systematic reviews of adult in-hospital populations evaluating EWS as either prognostic tools for deterioration or mortality, or as interventions implemented within track-and-trigger or rapid response systems. Primary study overlap was quantified using the corrected covered area (CCA). We synthesized evidence narratively and tabularly, emphasizing ED-related data.

**Results:**

From 502 records identified, 21 systematic reviews comprising 343 primary studies were included. We identified ED-relevant evidence in 12 reviews. In ED populations, EWS demonstrated moderate-to-good discrimination for mortality and clinical deterioration (National EWS area under the receiver operating characteristic curve (AUROC) values: 0.88 for 24-hour mortality, 0.86 for 48-hour mortality). Across ED and mixed acute-care cohorts, Early Warning Scores also showed fair performance for predicting ICU admission and composite adverse outcomes (AUROC ~0.70–0.75). In sepsis-focused analyses relevant to ED populations, quick Sequential Organ Failure Assessment (qSOFA) demonstrated low sensitivity but high specificity for 28/30-day mortality (sensitivity 0.41, 95% CI, 0.24–0.62; specificity 0.88, 95% CI, 0.81–0.92). The SOFA score showed high sensitivity and moderate specificity for in-hospital mortality (sensitivity 0.89, 95% CI, 0.88–0.90; specificity 0.69, 95% CI, 0.68–0.69). Evidence on the clinical impact of EWS implementation in ED and other emergency settings was limited and heterogeneous.

**Conclusion:**

In EDs, Early Warning Scores support early risk stratification and identification of patients at risk of deterioration, particularly for short-term mortality and escalation of care. Their impact on patient-important outcomes remains uncertain. Early Warning Scores should complement clinical judgment, triage systems, and structured escalation protocols. They should not be a standalone tool. Future research should prioritize standardized thresholds, transparent reporting, and rigorous comparative and implementation studies defining the role of EWS in emergency care.

## INTRODUCTION

Emergency departments (ED) manage patients with diverse and initially undifferentiated clinical presentations. Because decisions regarding triage, escalation of care, and patient disposition are often made before complete diagnostic information is available, clinicians increasingly rely on structured approaches to risk stratification. When implemented within structured rapid response or track and trigger systems, Early Warning Scores (EWS) facilitate early recognition of patient deterioration, prompting care escalation and timely appropriate clinical intervention.^1^ Early Warning Scores are based on routinely recorded physiological parameters, such as respiratory rate, heart rate, blood pressure, temperature, oxygen saturation, and level of consciousness.^2^

The evidence base evaluating Early Warning Scores encompasses a wide range of primary studies, including randomized and nonrandomized trials, as well as secondary research.^3^–^6^ Given the heterogeneity of this literature, several scoping reviews have attempted to map the field and describe the range of available tools and study designs.^7^–^9^ Existing primary and secondary studies address two closely related aspects of EWS use. One focuses on the predictive value of EWS and its ability to identify patients at risk of adverse outcomes, such as mortality or admission to the intensive care unit (ICU). Other studies evaluate the impact of EWS implementation on clinical outcomes and healthcare resource utilization, including mortality, length of hospital stay, and rates of serious adverse events.^10^–^13^

For the purposes of this overview of reviews, we distinguish between the EWS and the broader Early Warning System. The EWS refers to the numerical score derived from physiological observations. In contrast, an Early Warning System, often described as a track and trigger system (TTS), integrates the score with structured escalation protocols to ensure appropriate and timely clinical action. For example, within the National Early Warning Score (NEWS), a score of 5 or greater typically prompts an increased level of monitoring, while a score of 7 or greater triggers urgent clinical review or an emergency response.^14^ Although our primary analyses address the predictive performance of EWS, their real-world effectiveness is inherently linked to systematic clinical implementation within structured escalation pathways.

From a clinical and research perspective, substantial variability exists in the types of scores used, their modifications, threshold values, and the outcomes assessed. This heterogeneity complicates implementation in routine practice and limits comparability across studies and healthcare systems.^15^,^16^ As a result, despite the large volume of research, the overall strength and consistency of evidence supporting the effectiveness of EWS, particularly in improving patient outcomes, remain limited.^1^

Given this heterogeneity in study populations, score definitions, and reported outcomes, we conducted an overview of reviews to synthesize evidence from existing systematic reviews addressing EWS in adult in-hospital settings with special focus on EDs. This study design enables the summarization of the evidence from systematic reviews of different interventions for the same population and setting. This approach allows the exploration of research questions with a broader scope than those typically addressed in individual systematic reviews.^17^,^18^

**Population Health Research Capsule**

What do we already know about this issue?

*Early Warning Scores (EWS) use vital signs to identify clinical deterioration, but evidence on their emergency department (ED) performance and outcome impact is heterogeneous*.

What was the research question?

*How do EWS perform and affect outcomes in adult ED and inpatient populations?*

What was the major finding of the study?

*National Early Warning Score (NEWS) predicted ED mortality with AUROC 0.88 at 24h and 0.86 at 48h; implementation effects were uncertain*.

How does this improve population health?

*EWS may support earlier ED risk stratification, but must be embedded in triage, reassessment, and escalation pathways*.

The aim of this overview was to synthesize evidence from systematic reviews on the predictive performance and clinical impact of EWS and their modifications in adult ED and acute in-hospital populations, with particular attention to outcomes relevant to emergency care, including mortality, ICU or high-dependency unit (HDU) admission, cardiac arrest, serious adverse events, and length of stay.

## METHODS

This review was conducted in accordance with the Joanna Briggs Institute (JBI) methodology for umbrella reviews.^19^ Reporting followed the Preferred Reporting Items for Overviews of Reviews (PRIOR) statement.^20^ The study protocol was prospectively registered in the Open Science Framework (https://osf.io/gbevn/).

### Eligibility criteria

Eligibility criteria were defined using the PICOS framework (Population, Intervention, Comparison, Outcomes, Study design).

- **Population**: We included systematic reviews of adult hospitalized patients. Reviews focusing on pediatric or obstetric populations were excluded.
- **Intervention/Comparison**: We included reviews evaluating any type of EWS and its modifications, as well as reviews comparing different track and trigger or rapid response systems. We excluded reviews focusing exclusively on paediatric or obstetric EWS.
- **Outcome**: We summarized evidence on the predictive performance and clinical impact of EWS. The primary outcome was mortality; secondary outcomes were length of hospital stay (LOS), ICU, or HDU admission (including unplanned admission), in-hospital cardiac arrest, and serious adverse events. We also extracted review-level recommendations regarding the use of specific EWS.
- **Settings**: We considered only reviews of hospital-based settings. Reviews collecting evidence on prehospital settings were excluded.
- **Study design**: We selected the systematic reviews published in peer-reviewed journals. Systematic reviews were defined as studies that reported a clearly formulated research question and conducted searches in at least two databases. Scoping reviews, primary research studies, and conference abstracts were excluded.

### Information Sources and Search Strategy

An information specialist conducted a comprehensive search in May 2023 to identify systematic reviews related to EWS, using the CADTH filter for systematic reviews.^21^ An initial search was performed in MEDLINE (Ovid) using relevant keywords and index terms, followed by a refined search using all identified terms across MEDLINE (Ovid) and Epistemonikos. This database combination was chosen to optimize the retrieval of systematic reviews.^22^ No restrictions were applied regarding language or geographic location. However, no relevant non-English studies were identified. Reference lists of included reviews were also screened. Full search strategies are provided in Appendix A.

Retrieved records were imported into the Zotero (AGPL) reference management software tool and deduplicated using the systematic review accelerator.^23^ The search was updated in May 2025, with new records deduplicated against the original dataset using the same process.^24^

### Selection Process

Records were managed using Zotero (AGPL) and the PICO Portal. 25 Two reviewers (MP, JM) independently screened titles, abstracts, and full texts for eligibility. The screening process was piloted prior to formal screening. Disagreements were resolved by a third reviewer (AL).

### Data Collection Process

Data extraction was performed independently by two reviewers (AL, MP) using prepiloted Microsoft Excel forms. Extracted data included review title, year of publication, population characteristics, types of EWS and track and trigger systems, number and identity of included primary studies, methods of data analysis, reported heterogeneity, outcomes and conclusions, and review-level recommendations. Extraction tables are provided in Appendix B.

To quantify the overlap of primary studies across included reviews, citation matrices were constructed, and we calculated corrected covered areas (range, 0–100%). Overlap was classified as slight (0–5%), moderate (6–10%), high (11–15%), or very high (> 15%). Overlap was visualized using the GROOVE tool. When overlapping reviews reported discordant findings, priority was given to the most recent and methodologically robust review, with verification against primary studies where necessary.^26^,^27^ Reviews were additionally categorized by population, intervention, and comparison (PICOS/PIRDs), and we identified no systematic reviews with identical populations, interventions, comparisons, and settings (or index and reference tests).

### Risk of Bias Assessment

Two reviewers (AL, TF) independently assessed the risk of bias in included studies based on the JBI critical appraisal tool for systematic reviews.^26^ A third reviewer (MP) resolved conflicting decisions. The visualization was performed using Review Manager 5.4.^28^ Risk of bias in individual primary studies was not reassessed, as this was beyond the scope of this overview of reviews.

### Synthesis Methods

Findings were synthesized narratively, with the characteristics and key results of the included reviews summarized in tables. We recorded reported measures of heterogeneity. This overview provides a structured synthesis of existing systematic reviews, without involving reanalysis or meta-analysis of primary study data.

## RESULTS

The search yielded 502 records. After removing duplicate records, 348 records were screened at the title/abstract and full-text levels. The flow of records through the different phases of this overview of reviews is shown in the PRISMA 2020 flow diagram (Figure).^29^ The list of studies excluded based on the full-text screening is attached in Appendix C.

The overall corrected covered area was 2.03%, indicating slight overlap among the included reviews. Most pairs of reviews showed no or minimal overlap in primary studies, suggesting that the findings are unlikely to be substantially influenced by overlapping evidence. Overlap was visualized using the GROOVE tool (Appendix D).^27^

### Risk of Bias in Systematic Reviews

Risk of bias assessments for the included systematic reviews are presented in Table 1. Most reviews demonstrated high methodological quality, with 7–11 domains rated as low risk of bias using the JBI critical appraisal tool.

### Summary of Results

In total, 21 systematic reviews comprising 343 primary studies were included. The reviews evaluated the prognostic performance of EWS, the impact of their implementation as an intervention, or both. Key characteristics of the included reviews are summarized in Tables 2–4.

### Early Warning Scores in Emergency Department Settings

Among the 21 included systematic reviews, ED-specific or ED-relevant evidence was identified in 12 reviews. Three reviews focused specifically on adult ED populations: one systematic review of physiologically based early warning or track-and-trigger systems after triage in EDs; and one systematic review comparing qSOFA with hospital EWS in ED patients with suspected sepsis.^15^,^32^,^38^ One additional review focused on the ED and acute medical unit and was therefore considered directly relevant to acute unscheduled care. 36 Eight additional reviews included ED cohorts as part of mixed-setting populations, including combinations of ED, general ward, ICU, acute inpatient, or outside-ICU settings. These reviews were considered ED-relevant but not ED-specific, and their findings were interpreted cautiously because ED-specific estimates could not always be separated from those reported in other inpatient settings.^18^,^30^–^31^,^33^,^35^,^37^,^39^–^40^

### Included Tools and Their Characteristics

Across the included reviews, we identified more than 39 scoring tools, ranging from universal bedside Early Warning Scores (eg, NEWS/NEWS2 and MEWS variants) to sepsis screening tools (qSOFA, SOFA, Systemic Inflammatory Response Syndrome), condition-specific severity scores, triage scales, and other specialty tools.^15^,^31^,^37^,^40^,^41^,^46^ Key examples and inputs are summarized in Table 5. Most tools rely on core physiological observations (respiratory rate, heart rate, oxygen saturation, level of consciousness, blood pressure, and temperature), although some models additionally include laboratory parameters and comorbidity measures.^15^,^31^,^36^

### Predictive Performance of Early Warning Scores

This section summarizes the predictive performance of Early Warning Scores as reported in the included systematic reviews (Tables 2 and 4), focusing on area under the curve (AUROC/AUC), sensitivity, and specificity.

#### Mortality

Across ED and ward cohorts, Early Warning Scores generally show moderate-to-good discrimination for short-term mortality (AUC/AUROC commonly ~0.70–0.90). NEWS and MEWS typically demonstrate moderate discrimination in sepsis/infection cohorts, whereas qSOFA is generally more specific but less sensitive. In a sepsis-focused meta-analysis, pooled sensitivity/specificity were 0.89/0.69 for SOFA in predicting in-hospital mortality and 0.41/0.88 for qSOFA in predicting 28/30-day mortality. 37 Heterogeneity is noticeable across most studies, settings, and thresholds.^15^,^30^–^33^,^35^–^40^

#### Intensive Care Unit/High-Dependency Unit Admission

Evidence for predicting ICU/HDU admission is mixed. In ED sepsis and mixed-acuity cohorts, NEWS and MEWS show fair discrimination for ICU admission, with sensitivity varying by threshold. On general wards, discrimination for unplanned ICU admission is often moderate, but the evidence is mostly from heterogeneous observational studies.^15^,^31^,^32^,^35^,^37^–^41^

#### Cardiac Arrest

For in-hospital cardiac arrest, reported discrimination is moderate to good and depends strongly on the chosen thresholds. In one systematic review, CART (AUROC ~0.84) outperformed MEWS (~0.78), and NEWS also showed good short-term performance.^46^ One study noted that cardiac arrest was the least examined endpoint.^31^

#### Serious Adverse Events

In ED settings, combined endpoints of ICU admission or death were predicted with AUROCs around 0.70–0.75 for NEWS and 0.73–0.76 for MEWS models.^36^,^38^ In infection cohorts outside the ICU, NEWS shows moderate discrimination for ICU admission (AUC ~0.71), with sensitivity ~0.71 and specificity ~0.55.^37^,^38^,^40^ In COVID-19 cohorts, NEWS2 shows good discrimination for deterioration outcomes (AUC ~0.82) with pooled sensitivity ~0.82 at commonly used thresholds (e.g., ≥5). 41

#### Predictive Performance in the Emergency Department

Emergency department-specific reviews support the relevance of EWS for risk stratification, particularly for early mortality, in-hospital mortality, hospital admission, and ICU admission. Emergency department-specific quantitative evidence came from the systematic review and meta-analysis, where NEWS showed good discrimination for 24-hour mortality (AUROC 0.88) and 48-hour mortality (AUROC 0.86), with lower but still acceptable discrimination for in-hospital mortality (AUROC 0.77). The NEWS also showed adequate predictive ability for hospital and ICU admission, although the findings were limited by heterogeneity in scores and thresholds.^32^

This ED-focused evidence was consistent with broader ED and acute-care review, which found that EWS and track-and-trigger systems used after ED triage can predict mortality and ICU admission, but that the evidence is mainly observational and heterogeneous.^15^ In ED and acute medical unit populations, NEWS and MEWS generally showed favorable prognostic performance, although score performance varied by subgroup and clinical context.^36^

### Impact of Early Warning Scores Implementation on Clinical Outcomes

This section synthesizes evidence on whether implementing EWS within track and trigger/ rapid response systems is associated with changes in clinical outcomes (Tables 3 and 4).

#### Mortality

Evidence on mortality reduction after EWS implementation is mixed. A meta-analysis reported reduced unexpected in-hospital mortality after EWS implementation, but underlying studies were largely non-randomized and heterogeneous. Other reviews similarly describe a pattern of variable effects, with frequent risk of bias and inconsistent findings across settings.^1^,^18^,^42^,^43^,^45^

#### Intensive Care Unit/High-Dependency Unit Admission

Effects of Early Warning Scores/Rapid Response System implementation on ICU/HDU admissions are inconsistent. Randomized evidence does not show a clear reduction in unplanned ICU admissions, while before-and-after studies suggest changes in escalation patterns rather than consistent reductions.^1^,^18^,^42^,^43^,^45^

#### Cardiac Arrest

Effects on in-hospital cardiac arrest after EWS implementation are inconsistent. Some before-and-after studies report reductions, whereas others report no change or increases; randomized evidence does not demonstrate a consistent reduction.^1^,^18^,^42^,^43^,^45^

#### Serious Adverse Events

Definitions of serious adverse events varied widely across studies, limiting synthesis. Some ward and surgical cohorts reported improvements in selected serious adverse events endpoints after implementing EWS observation/escalation protocols, but results were inconsistent and generally low certainty.^1^,^18^,^42^,^45^

#### Length of Stay

Across reviews, the effect of EWS/Rapid Response System implementation on hospital LOS was small or inconsistent. Randomized evidence suggests little to no effect, and observational findings are mixed and likely influenced by differences in baseline severity.^1^,^18^

#### Implementation Evidence in Emergency Departments

Evidence on ED implementation was more limited than evidence on predictive performance. A review of EWS use after ED triage found only limited comparative evidence, including one nonrandomized controlled trial suggesting changes in patient management but no clear reduction in adverse events.^15^ A further mixed-setting review reported improved outcomes after implementation, but without ED-specific pooled estimates.^18^

### Summary of Recommendations

Across included reviews, practice-oriented recommendations emphasized limitations, appropriate use, and implementation considerations. In suspected sepsis or infection, reviews consistently flagged that EWS should not be used in isolation for prognostication or high-stakes disposition decisions; instead, they should support ongoing monitoring and a structured clinical response.^33^,^40^,^41^ Where multiple scores/criteria were compared, some reviews recommended combining criteria to balance sensitivity and specificity.^35^,^37^,^39^ Implementation-focused reviews report the importance of linking scoring to clear escalation pathways (including outreach/Rapid Response System) and tailoring to local resources.^43^,^44^ In ED settings, this also implies that EWS should be integrated into triage, reassessment, and escalation workflows rather than interpreted as isolated single time-point measurements.^15^,^38^,^40^ Several reviews called for more rigorous prospective evaluations of EWS/Rapid Response Systems (including multicenter and cluster-randomized designs), ideally using a single standardized score (eg, NEWS/NEWS2) and agreed outcome definitions, with reporting of adherence to escalation protocols, to enable stronger synthesis.^1^,^18^,^32^,^42^,^43^,^45^,^46^ Recurrent priorities also included validation in specific patient subgroups and settings (older adults and other high-risk groups; low-resource settings), assessment of health-economic impact, and further tool development/improvement.^15^,^16^,^30^,^31^,^34^,^36^,^38^ Detailed review-level recommendations are provided in Appendix B.

## DISCUSSION

### Interpretation

In this overview of reviews, we synthesized secondary evidence on the predictive performance and clinical impact of EWS across ED and acute-care settings. Overall, EWS-based tools demonstrated moderate-to-good discrimination for identifying patients at risk of short-term deterioration, particularly early mortality and need for escalation of care in ED populations. However, evidence that implementation of EWS-based systems improves patient-important outcomes remained limited and inconsistent across both ED and inpatient settings.

### Summary of Main Findings

Most often studied scores, such as NEWS/NEWS2 and MEWS, discriminate patients at risk of in-hospital or 30-day mortality with AUROCs typically ranging from 0.70 to 0.80 outside the ICU, with somewhat higher values in acute medical and ED cohorts, while sepsis-focused scores show variable sensitivity–specificity.^31^–^33^,^35^,^37^–^41^ Intervention reviews of rapid response or track and trigger systems based on EWS show, at best, small and inconsistent reductions in unexpected deaths, ICU admissions, cardiac arrests, or length of stay.^1^,^18^,^42^–^46^ Together, these findings support viewing EWS as one component of a robust rapid response system rather than a standalone solution for preventing unexpected deterioration.

Across general ward and ED settings, EWS achieved better discrimination for mortality and deterioration than single-parameter criteria, while still generating false positives.^15^,^31^,^32^,^37^–^41^,^44^ In ED populations, EWS-based tools predicted in-hospital or 30-day mortality with AUROCs ranging between 0.77 and 0.87. In contrast, qSOFA offered higher specificity but considerably lower sensitivity, meaning many deteriorating patients would be missed if it were used as a single trigger.^15^,^32^,^37^–^39^ Meta-analyses confirmed that no bedside score combining vital signs and simple clinical variables offers both high sensitivity and high specificity for mortality; SOFA had high sensitivity and moderate specificity, whereas qSOFA and NEWS traded higher specificity for reduced sensitivity.^30^,^33^,^35^,^37^–^39^

Across included implementation reviews, EWS generally show favorable trends (eg, improved monitoring and escalation). The effects on mortality after implementation remain uncertain and highly variable across settings and study designs. Randomized evidence suggests little or no difference in hospital mortality and serious adverse events.^1^,^18^,^42^–^46^

Considerable heterogeneity in patient populations, inclusion criteria, outcome definitions and threshold values was a consistent finding in almost all reviews, with I^2^ statistics frequently exceeding 75–90% for sensitivity and specificity estimates.^18^,^31^,^33^,^35^,^37^–^39^,^44^,^46^ This variability reflects differences in case mix and processes between various clinicians, together with inconsistent reporting and variable adherence to escalation protocols, which limit the interpretability of pooled estimates.^15^,^44^,^31^,^32^,^37^–^39^,^18^,^46^

### Comparison to Previous Studies

We address a specific gap in the literature by synthesizing secondary evidence on predictive performance and clinical outcomes for adult in-hospital populations, with a focus on ED settings. Previous systematic reviews have typically focused on either particular tools, specific conditions, or single care settings, such as the ED or ICU.^15^,^18^,^30^,^32^,^35^–^39^,^42^–^46^ By including 21 reviews and 343 primary studies, we provide a higher-level view of how EWS function within contemporary RRS and where the main uncertainties lie. A recent rapid umbrella review of EWS also reported substantial heterogeneity in both results and evidence levels across EWS reviews and concluded that no general recommendation can currently be made for or against widespread EWS implementation, while suggesting that implementation may be most appropriate in high-risk settings.^47^ Our overview extends this knowledge by focusing on adult inpatients, quantifying primary study overlap and integrating both prognostic and implementation outcomes, thereby providing a more detailed map of where evidence is relatively robust and where it remains fragmentary. Importantly, our overview also separates two questions that are often discussed together in the literature: the prognostic performance of EWS and the clinical impact of their implementation within escalation systems.

The Cochrane review^1^ and subsequent systematic reviews concluded that EWS-based RRSs may reduce cardiopulmonary arrests and unexpected ICU admissions, but have uncertain effects on in-hospital mortality because of nonrandomized designs.^1^,^18^,^42^–^46^ This more cautious interpretation contrasts with some earlier reviews and meta-analyses that suggested more favorable effects on mortality or cardiac arrest, and likely reflects the use of stricter methodological criteria and greater attention to heterogeneity in more recent syntheses, particularly the updated Cochrane review. Earlier EWS reviews also highlighted similar methodological problems, including small single-center cohorts, inconsistent thresholds, and limited adjustment for sampling, which are also evident in the more recent intervention literature summarized in this overview.^18^,^42^–^46^

In ED populations, previous systematic reviews have reported that vital signs-based scores, such as NEWS and MEWS, outperform traditional triage scales for predicting short-term mortality or ICU admission; however, the incremental value of any single score is modest and setting-dependent.^15^,^32^,^36^ Our synthesis aligns with recent sepsis-focused meta-analyses showing that qSOFA has high specificity but low sensitivity, whereas NEWS and SIRS offer better sensitivity at the cost of more false positives, and that SOFA-based approaches provide the highest overall prognostic accuracy at the expense of resources.^30^,^33^,^35^,^37^–^39^ These reviews consistently show that no score simultaneously achieves both high sensitivity and high specificity, which our overview confirms across a broader inpatient context.^30^,^33^,^35^,^37^–^39^

### Strengths

Our research highlights the need for synthesized evidence on various EWS and track and trigger systems, which is currently lacking in both the research literature and clinical practice. The robust findings, supported by a standardized methodological approach, cover a high quality of systematic reviews. The results offer valuable insights for clinicians, researchers, and policymakers on the efficiency of EWS and its modifications.

### Clinical Implications

For emergency medicine practice, the available evidence supports the use of structured Early Warning Scores (particularly NEWS/NEWS2 and MEWS) as adjuncts to clinical assessment for identifying patients at risk of deterioration. In ED settings, EWS may help support triage, serial reassessment, escalation of care, and disposition decisions by providing a standardized assessment of physiological instability using routinely collected vital signs.

However, the moderate discriminatory performance of currently available scores and the substantial heterogeneity across studies indicate that EWS should not be used as standalone decision-making tools. In suspected infection or sepsis, qSOFA generally provides higher specificity but lower sensitivity than NEWS/NEWS2, meaning that reliance on qSOFA alone may fail to identify a substantial proportion of deteriorating patients. Although SOFA-based approaches demonstrate higher sensitivity, their practical applicability in the ED may be limited by the need for laboratory data and greater complexity.

The effectiveness of EWS in emergency care likely depends not only on score performance itself but also on integration with clinical judgment, established triage systems, staffing resources, repeated patient assessment, and clearly defined escalation pathways. This may be particularly important in crowded or resource-constrained ED environments where patient acuity and clinical priorities can change rapidly.^39^–^41^

### Research Implications

The limited and low-certainty effect of EWS-based tools on mortality highlights the need for methodologically robust trials that compare different escalation strategies and support comparability.^1^,^3^,^4^,^18^,^42^–^45^ Future studies should adopt a standardized set of outcomes–including early and in-hospital mortality, unplanned ICU admission, in-hospital cardiac arrest, well-defined serious adverse events, and patient-centered outcomes. We recommend clearly reporting decision threshold levels in studied scores to prevent high heterogeneity.^31^–^33^,^37^,^38^,^43^,^44^,^46^ We also recommend focusing future studies on healthcare personnel’s compliance with EWS-based systems.^18^,^46^

In emergency medicine and sepsis, comparative prognostic studies of NEWS/NEWS2, qSOFA, SOFA, and emerging machine learning models should focus on the incremental value over existing triage systems and clinician gestalt, particularly in high-risk subgroups such as older adults and patients with immunosuppression.^15^,^30^,^32^,^35^,^37^–^39^,^48^,^49^ Implementation research is also needed to understand how staffing, training, electronic alert design and local escalation protocols influence the effectiveness of EWS in real-world settings, including low and middle-income countries where case mix and resources differ substantially.^7^–^9^,^15^,^18^,^30^,^35^,^36^,^39^,^46^

## LIMITATIONS

This review is limited by heterogeneity across included systematic reviews, most of which synthesized observational studies with varying thresholds, outcome definitions, and implementation strategies. We did not formally grade certainty of evidence because substantial clinical and methodological diversity would have made such ratings difficult to interpret. Risk of bias in individual primary studies was not reassessed, and our findings therefore depend on the quality and reporting of the included reviews. In addition, implementation effects are difficult to attribute solely to EWS due to co-interventions, time-related changes in care, and the predominance of nonrandomized study designs, while agreement between predicted and observed risk and other clinical utility measures were inconsistently reported.

## CONCLUSION

In ED settings, Early Warning Scores, particularly NEWS/NEWS2 and MEWS, provide clinically useful support for early risk stratification and identification of patients at risk of deterioration, especially for short-term mortality and escalation of care. However, their overall impact on patient-important outcomes remains uncertain, and current evidence supporting implementation effectiveness in ED workflows is limited and heterogeneous.

In suspected infection/sepsis cohorts, NEWS/NEWS2 (and, where evaluated, MEWS) generally show higher sensitivity than qSOFA at commonly used thresholds, whereas qSOFA tends to be more specific. Overall, Early Warning Scores can help reduce the risk of unexpected deterioration, but they must be embedded in clear escalation protocols and supported by adequate staffing, resources, and clinical judgment, rather than used as standalone solutions.

## Supplementary Information











## Figures and Tables

**Figure:**
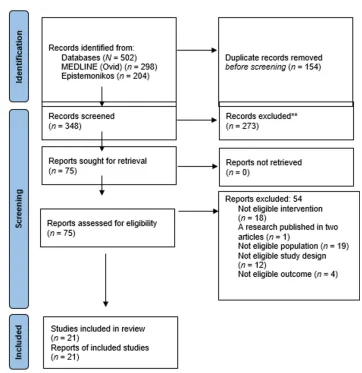
PRISMA 2020 flow diagram showing the identification, screening, and inclusion of systematic reviews in this review.^29^ According to: Page MJ, et al. BMJ 2021;372:n71. doi:10.1136/bmj.n71. *PRISMA*, Preferred Reporting Items for Systematic Reviews and Meta-Analyses.

**Table 1:**
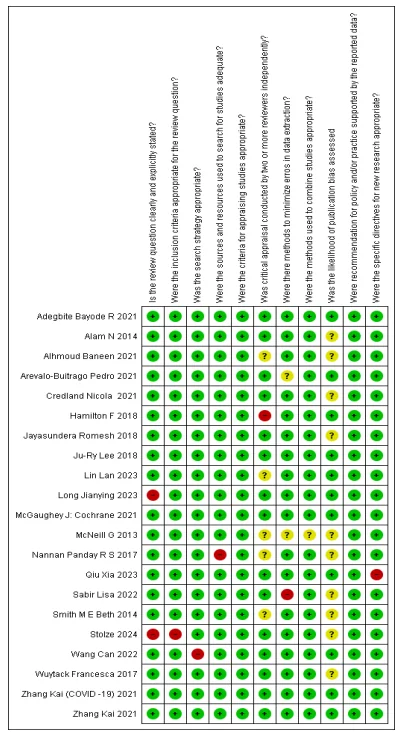
Risk of bias assessment of included systematic reviews using the Joanna Briggs Institute critical appraisal tool. Green (+) indicates a “Yes” response (low risk of bias), red (−) indicates a “No” response (high risk of bias), and yellow (?) indicates an “Unclear” response (unclear risk of bias).

**Table 2:** Characteristics of included systematic reviews evaluating the predictive performance of Early Warning Scores (emergency department settings marked in bold).

Author, year, reference	N (included primary studies)	N (number of patients)	Population	Settings	Assessed score	Outcomes
Adegbite (2021)^30^	24	27,237	Adult and adolescent patients (≥15 years old) with suspected infection or sepsis	**ED**, GW, ICU	EWS, MEWS, qSOFA, SIRS, UVA	Acute organ dysfunction, Mortality
Alhmoud (2021)^31^	103	3,805,052	Adult and adolescent patients (≥16 years old)	**ED**, ICU, MD, SD	EWS, HEWS, HOTEL, MEWS, NEWS, NEWS 2, SOS, TREWS, ViEWS, WORTHING	Cardiac arrest, ICU admission, Mortality
Arévalo-Buitrago (2021)^32^	9	165,580	Adult patients (≥18 years old)	**ED**	MEWS, NEWS, REMS, SEWS, TREWS, ViEWS	ICU admission, Hospital admission, Mortality
Hamilton (2018)^33^	6	4,298	Adult patients (≥18 years old) with sepsis	**ED**, MD	EWS, MEWS	Short-term mortality (in-hospital or 28–30-day mortality)
Lan (2024)^35^	32	55,088	Adult patients with sepsis	**ED**, ICU	MEWS, SIRS, SOFA	28-day mortality, 30-day mortality, In-hospital mortality
Nannan Panday* (2017)^36^	42	166,344	Adult and adolescent patients (≥16 years old)	**ED**, ICU	APACHEII, CCI, CRB-65, CURB-65, CREWS, ESI, EWS, GAP, MEDS, MEES, MEWS, NEWS, PIRO, PSI, qSOFA, RAPS, REMS, RTS, SEWS, SCS, SIRS, PEDS, THERM, ViEWS, WPS	ICU admission, Mortality
Xia Qiu (2023)^37^	57	227,726	Adults (two articles did not specify the age range)	**ED**, GW, ICU	NEWS, qSOFA, SIRS, SOFA	7-day mortality, 10-day mortality,14-day mortality, 28-day mortality, 30-day mortality, In-hospital mortality, ICU admission, Sepsis
Sabir (2022)^38^	13	403 865	Adult patients with suspected sepsis	ED	EWS, MEWS, NEWS, NEWS 2, qSOFA	ICU admission, Mortality
Wang (2022)^39^	26	62,338	Patients with suspected sepsis	ED, Acute inpatient department, Outside the ICU	NEWS, qSOFA, SIRS	28-day mortality, 30-day mortality, In-hospital mortality
Zhang (2021)^40^	21	107,008	Adult patients (≥18 years old) with infection or sepsis	ED, GW, Outside the ICU	NEWS, NEWS 2	In-hospital mortality, 30-day mortality, ICU admission
Zhang (2021)^41^	18	6,922	Adult patients with confirmed cases of SARS-CoV-2 infection	Mostly general ward, not always reported	NEWS, NEWS 2, qSOFA	In-hospital mortality, 30-day mortality, ICU admission
Long (2023)^34^	29	1,039,992	Adult patients (≥18 years old) hospitalized in the ICU for more than 24 h	ICU	EWS, MEWS, NEWS	ICU readmission after ICU discharge (follow-up 24 h to 30 days, depending on primary study)

* This study is self-described as a “narrative review” in the title. However, its methodology fulfilled our inclusion criteria for systematic reviews, as it conducted comprehensive searches in multiple databases, applied explicit eligibility criteria, and presented a transparent selection process. Therefore, it was retained in our analysis.

*APACHE II*, Acute Physiology and Chronic Health Evaluation II; *AWTTS*, Aggregate Weighted Track and Trigger Systems; *BEWS*, Bispebjerg Early Warning Score; *CART*, Cardiac Arrest Risk Triage; *CCI*, Charlson Comorbidity Index; *CCO*, Critical Care Outreach; *CRB-65*, Confusion, Urea nitrogen, Respiratory rate, Blood pressure and age 65 years and over; *CREWS*, Chronic Respiratory Early Warning Score; *CURB-65*, Confusion, Urea nitrogen, Respiratory rate, Blood pressure and age 65 years and over; *ED*, Emergency Department; *ESI*, Emergency Severity Index; *EWS*, Early Warning Score; *GAP*, Glasgow, Age, Arterial pressure; *GW*, General Ward; *HEWS*, Hamilton Early Warning Score; *HOTEL*, Hypotension, low-Oxygen, low-Temperature, abnormal ECG, Loss of independence; *ICU*, Intensive Care Unit; *LOS*, Length of Stay; *MD*, Medical Department; *MEDS*, Mortality in the Emergency Department Sepsis score; *MEES*, Mainz Emergency Evaluation Score; *MEWS*, Modified Early Warning Score; *mREMS*, Modified Rapid Emergency Medicine Score; *N*, Number of included studies; *NEWS*, National Early Warning Score; *NEWS-L*, National Early Warning Score–Lactate; *PEDS*, Prince of Wales Emergency Department Score; *PIRO*, Predisposition, Infection, Response and Organ failure; *PSI*, Pneumonia Severity Index; *qSOFA*, quick Sequential Organ Failure Assessment; *RAPS*, Rapid Acute Physiology Score; *REMS*, Rapid Emergency Medicine Score; *RTS*, Revised Trauma Score; *SCS*, Simple Clinical Score; *SD*, Surgical Department; *SEWS*, Standardised Early Warning Score; *SIRS*, Systemic Inflammatory Response Syndrome; *SOFA*, Sequential Organ Failure Assessment; *SOS*, Search Out Severity; *TEWS*, Triage Early Warning Score; *TREWS*, Targeted real-time early warning score; *THERM*, The Resuscitation Management Score; *UVA*, Universal Vital Assessment; *ViEWS*, VitalPAC Early Warning Score; *ViEWS-L*, VitalPAC Early Warning Score–Lactate; *WPS*, Worthing Physiological Scoring System.

**Table 3:** Characteristics of included systematic reviews assessing the implementation of Early Warning Scores.

Author, year, reference	N (included primary studies)	N (number of patients)	Population	Settings	Intervention	Comparison	Outcomes
Alam (2014)^42^	7	486,237	Adult and adolescent patients (≥ 16 years old)	GW, MD	MEWS	No MEWS + CCO present vs. no MEWS + no CCO	Documentation of physiological parameters, Cost-effectiveness, Cardiopulmonary arrest, HDU and ICU admission, ICU mortality, LOS, Mortality, SAE
Lee et al. (2018)^43^	5	11,753	Adult patients (≥18 years old)	GW with rapid response team	AWTTS, EWS, MEWS, NEWS	Not specified	Cardiac arrest, ICU admission, In-hospital mortality
McGaughey (2021)^1^	11	666,131	Adult patients at risk of clinical deterioration	Acute in-hospital settings	EWS, MEWS, NEWS, PAR and single parameter calling criteria as defined by RRS	Not specified	Admission to ICU, Cardiac arrest, Death, LOS, Readmission, Respiratory arrest, Unexpected death
McNeill (2013)^44^	4	677,025	Adult patients	In-hospital settings	EWS, MEWS	Not specified	Cardiac arrest rates, Hospital survival, In-hospital mortality, ICU mortality, Length of ICU stay, LOS, Unplanned ICU admissions
Stolze (2024)^45^	8	118,860	Surgical adult patients (≥18 years old)	MD, High-intensity monitoring area	EWS	Not specified	Cardiopulmonary arrest, Emergency surgery, In-hospital mortality, LOS, Unplanned ICU admission

*AWTTS*, Aggregate Weighted Track and Trigger Systems; *CCO*, Critical care outreach; *HDU*, high dependency unit; *ICU*, intensive care unit; *MEWS*, Modified Early Warning Score; *MD*, medical department; *PAR*, Patient At Risk score; *NEWS*, National Early Warning Score; *RRS*, rapid response systems; *SAE*, serious adverse events.

**Table 4:** Characteristics of included systematic reviews – both systematic reviews evaluating predictive values and systematic reviews assessing the impact of Early Warning Scores (and its modification) intervention.

Author, year, reference	N (included primary studies)	N (number of patients)	Population	Settings	Intervention, Comparison, intervention/control; Type of Test	Outcomes
Credland (2021)^18^	5	74,000	Adult patients	ED, GW, SD	EWS, NEWS	Cardiopulmonary arrest, HDU admission, Hospital admission, ICU admission, LOS, Mortality, Observation frequency, SAE
Wuytack (2017)^15^	36	45,221	Adult patients following initial triage	ED	BEWS, EWS, MEWS, MEWS plus, mREMS, NEWS, NEWS-L, TEWS, ViEWS, ViEWS-L	Death, Critical illness, Economic measures, Extent of use, ICU admission, LOS, Negative predictive value for adverse outcome/critical illness criterion, Number and type of clinical guidelines, Types of Early Warning Scores or TTS in use, Sensitivity/ Specificity for adverse outcome/critical illness criterion, Strategies and methods to evaluate education programmes, Positive predictive value for adverse outcome/critical illness criterion, Types of education programmes
Jayasundera (2018)^16^	5	5,321	Adult patients (≥ 65 years old) with sepsis and/or acute cardiovascular event	Acute hospital	EWS, MEWS	LOS, Morbidity, Mortality
Smith (2014)^46^	21	757,028 + 2 studies with NR	Adult patients	GW, SD	CART, EWS, NEWS, VIEWS	30-day mortality, 48-hour mortality, Cardiac arrest or pulmonary arrest Cardiovascular events, ICU admission, LOS, No. of days on a ventilator, Respiratory failure, RRT activation, Vasopressors use

*BEWS*, Bispebjerg Early Warning Score; *CART*, Cardiac Arrest Risk Triage; *GW*, general ward; *HDU*, high dependency unit; *ICU*, intensive care unit; *LOS*, length of stay; *MEWS*, Modified Early Warning Score; *NR*, Not reported; *SAE*, Serious Adverse Events; *mREMS*, Modified Rapid Emergency Medicine Score; *NEWS*, National Early Warning Score; *NEWS-L*, National Early Warning Score–Lactate; *RRT*, rapid response team; *SD*, surgical department; *TEWS*, Triage Early Warning Score; *ViEWS*, VitalPAC Early Warning Score; *ViEWS-L*, VitalPAC Early Warning Score–Lactate.

**Table 5:** Selected tools reported in included systematic reviews and their main characteristics.

Tool*	Simple inputs	Complex observation	Settings
Acute Physiology and Chronic Health Evaluation II (APACHE II)	RR, HR, Temp, Consciousness (GCS), FiO2, MAP, Age	Yes (ABG, Sodium, Potassium, Creatinine, Haematocrit, WBC and comorbidity input)	ICU
Cardiac Arrest Risk Triage (CART)	RR, HR, DBP, Age	No	ED/Wards
Confusion, Urea, Respiratory rate, Blood pressure and age 65 years and over (CURB65/CRB65)	RR, HR, Confusion, Age	Yes (Labs; BUN - only in CURB65)	ED
Emergency Severity Index (ESI)	RR, HR, SpO2, Consciousness	No (Expected clinical judgment)	ED
Charlson Comorbidity Index (CCI)	Age	Yes (Comorbidity)	Wards
Modified Early Warning Score (MEWS)	RR, HR, SBP, Temp, Consciousness (AVPU)	No	ED/Wards
Mortality in the Emergency Department Sepsis score (MEDS)	RR, SpO2, Consciousness (AVPU), BP, Age	Yes (Labs and comorbidity)	ED
National Early Warning Score (NEWS)	RR, SpO_2_, O_2_ supplement, HR, SBP, Temp, Consciousness (AVPU)	No	ED/Wards
National Early Warning Score 2 (NEWS2)	RR, SpO_2_, O_2_ supplement, HR, sBP, Temp, Refined consciousness (cAVPU), SpO_2_ scoring (incl. Scale 2 for hypercapnic risk)	No	ED/Wards
quick Sequential Organ Failure Assessment (qSOFA)	RR, SBP, mental status (GCS/AVPU)	No	ED
Rapid Emergency medicine score (REMS)	Age, GCS, MAP, HR, RR, SpO_2_	No	ED/non-surgical
Revised Trauma Score (RTS)	RR, sBP, GCS	No	Trauma
Glasgow, Age, Arterial pressure (GAP)	sBP, GCS, Age,	No	Trauma
Sequential Organ Failure Assessment (SOFA)	MAP, GCS, Urine output, Vasopressor use	Yes (ABG, Plt, bilirubin, creatinine)	Sepsis/ICU
Systemic Inflammatory Response Syndrome (SIRS)	RR, Temperature, HR	Labs (PaCO_2_)	Sepsis/ED
ViEWS/HEWS/TEWS/SEWS	NEWS/MEWS like sets with local calibrations	No labs	Wards/ED

* Complete list with detailed characteristics described in Appendix E.

*ABG*, arterial blood gases; *cAVPU*, confusion, Alert, Voice, Pain, Unresponsive; *BUN*, blood urea nitrogen; *CURB-65*, Confusion, Urea nitrogen, Respiratory rate, Blood pressure and age 65 years and over; *HR*, heart rate; *GCS*, Glasgow Coma Scale; *ICU*, intensive care unit; *Plt*, platelet count; *MAP*, mean arterial pressure; *RR*, respiratory rate; *sBP*, systolic blood pressure; *SpO*_2_, peripheral oxygen saturation.

## References

[1] McGaughey J, Fergusson DA, Van Bogaert P (2021). Early Warning Systems and rapid response systems for the prevention of patient deterioration on acute adult hospital wards. Cochrane Database Syst Rev.

[2] Holland M, Kellett J (2023). The United Kingdom’s National Early Warning Score: Should everyone use it? A narrative review. Intern Emerg Med.

[3] Haegdorens F, Van Bogaert P, Roelant E (2018). The introduction of a rapid response system in acute hospitals: a pragmatic stepped wedge cluster randomised controlled trial. Resuscitation.

[4] Jeddian A, Hemming K, Lindenmeyer A (2016). Evaluation of a critical care outreach service in a middle-income country: a stepped wedge cluster randomized trial and nested qualitative study. J Crit Care.

[5] Aitken LM, Chaboyer W, Vaux A (2015). Effect of a 2-tier rapid response system on patient outcome and staff satisfaction. Aust Crit Care.

[6] Menon VP, Prasanna P, Edathadathil F (2018). A quality improvement initiative to reduce “out-of-ICU” cardiopulmonary arrests in a tertiary care hospital in India: A 2-year learning experience. Qual Manag Health Care.

[7] Esmaeilzadeh S, Lane CM, Gerberi DJ (2022). Improving in-hospital patient rescue: What are studies on early warning scores missing? A scoping review. Crit Care Explor.

[8] Muralitharan S, Nelson W, Di S (2021). Machine learning-based early warning systems for clinical deterioration: Systematic scoping review. J Med Internet Res.

[9] Wood C, Chaboyer W, Carr P (2019). How do nurses use early warning scoring systems to detect and act on patient deterioration to ensure patient safety? A scoping review. Int J Nurs Stud.

[10] Chiu YD, Villar SS, Brand JW (2020). Logistic Early Warning Scores to predict death, cardiac arrest or unplanned intensive care unit re-admission after cardiac surgery. Anaesthesia.

[11] Smith GB, Prytherch DR, Meredith P (2013). The ability of the National Early Warning Score (NEWS) to discriminate patients at risk of early cardiac arrest, unanticipated intensive care unit admission, and death. Resuscitation.

[12] Hammer M, Grabitz SD, Teja B (2021). A tool to predict readmission to the intensive care unit in surgical critical care patients—The RISC score. J Intensive Care Med.

[13] Chen J, Ou L, Flabouris A (2016). Impact of a standardized rapid response system on outcomes in a large healthcare jurisdiction. Resuscitation.

[14] Smith GB, Redfern OC, Pimentel MA (2019). The National Early Warning Score 2 (NEWS2). Clin Med (Lond).

[15] Wuytack F, Meskell P, Conway A (2017). The effectiveness of physiologically based early warning or track and trigger systems after triage in adult patients presenting to emergency departments: a systematic review. BMC Emerg Med.

[16] Jayasundera R, Neilly M, Smith TO (2018). Are Early Warning Scores useful predictors for mortality and morbidity in hospitalised acutely unwell older patients? A systematic review. J Clin Med.

[17] Pollock M, Fernandes RM, Becker LA, Higgins JP, Thomas J, Chandler J (2024). Chapter V Overviews of reviews. Cochrane Handbook for Systematic Reviews of Interventions. Version 6.5.

[18] Credland N, Dyson J, Johnson MJ (2021). Do early warning track and trigger tools improve patient outcomes? A systematic synthesis without meta-analysis. J Adv Nurs.

[19] Aromataris E, Lockwood C, Porritt K (2024). JBI Manual for Evidence Synthesis.

[20] Gates M, Gates A, Pieper D (2022). Reporting guideline for overviews of reviews of healthcare interventions: development of the PRIOR statement. BMJ.

[21] Canadian Agency for Drugs and Technologies in Health (2024). CADTH Search Filters Database.

[22] Goossen K, Hess S, Lunny C (2020). Database combinations to retrieve systematic reviews in overviews of reviews: a methodological study. BMC Med Res Methodol.

[23] Clark J, Glasziou P, Del Mar C (2020). A full systematic review was completed in 2 weeks using automation tools: a case study. J Clin Epidemiol.

[24] Bramer W (2019). Serving evidence syntheses: improving literature retrieval in systematic reviews. PhD dissertation.

[25] Portal PICO https://picoportal.org/support/.

[26] Aromataris E, Fernandez R, Godfrey CM (2015). Summarizing systematic reviews: Methodological development, conduct and reporting of an umbrella review approach. JBI Evid Implement.

[27] Bracchiglione J, Meza N, Bangdiwala SI (2022). Graphical representation of overlap for Overviews: GROOVE tool. Res Synth Methods.

[28] (2020). Review Manager (RevMan) [computer program]. Version 5.4.

[29] Page MJ, McKenzie JE, Bossuyt PM (2021). The PRISMA 2020 statement: an updated guideline for reporting systematic reviews. BMJ.

[30] Adegbite BR, Edoa JR, Ndzebe Ndoumba WF (2021). A comparison of different scores for diagnosis and mortality prediction of adults with sepsis in low-and-middle-income countries: a systematic review and meta-analysis. eClinicalMedicine.

[31] Alhmoud B, Bonnici T, Patel R (2021). Performance of universal early warning scores in different patient subgroups and clinical settings: A systematic review. BMJ Open.

[32] Arévalo-Buitrago P, Morales-Cané I, Olivares Luque E (2021). Predictive power of early-warning scores used in hospital emergency departments: A systematic review and meta-analysis. Emergencias.

[33] Hamilton F, Arnold D, Baird A (2018). Early warning scores do not accurately predict mortality in sepsis: a meta-analysis and systematic review of the literature. J Infect.

[34] Long J, Wang M, Li W (2023). The risk assessment tool for intensive care unit readmission: a systematic review and meta-analysis. Intensive Crit Care Nurs.

[35] Lan L, Zhou M, Chen X (2024). Prognostic accuracy of SOFA, MEWS, and SIRS criteria in predicting the mortality rate of patients with sepsis: a meta-analysis. Nurs Crit Care.

[36] Nannan Panday RS, Minderhoud TC, Alam N (2017). Prognostic value of early warning scores in the emergency department (ED) and acute medical unit (AMU): A narrative review. Eur J Intern Med.

[37] Qiu X, Lei YP, Zhou RX (2023). SIRS, SOFA, qSOFA, and NEWS in the diagnosis of sepsis and prediction of adverse outcomes: a systematic review and meta-analysis. Expert Rev Anti Infect Ther.

[38] Sabir L, Ramlakhan S, Goodacre S (2022). Comparison of qSOFA and hospital early warning scores for prognosis in suspected sepsis in emergency department patients: a systematic review. Emerg Med J.

[39] Wang C, Xu R, Zeng Y (2022). A comparison of qSOFA, SIRS and NEWS in predicting the accuracy of mortality in patients with suspected sepsis: a meta-analysis. PLoS One.

[40] Zhang K, Zhang X, Ding W (2021). National Early Warning Score does not accurately predict mortality for patients with infection outside the intensive care unit: a systematic review and meta-analysis. Front Med (Lausanne).

[41] Zhang K, Zhang X, Ding W (2021). The prognostic accuracy of National Early Warning Score 2 on predicting clinical deterioration for patients with COVID-19: a systematic review and meta-analysis. Front Med (Lausanne).

[42] Alam N, Hobbelink EL, van Tienhoven AJ (2014). The impact of the use of the Early Warning Score (EWS) on patient outcomes: a systematic review. Resuscitation.

[43] Lee JR, Kim EM, Kim SA (2020). A systematic review of early warning systems’ effects on nurses’ clinical performance and adverse events among deteriorating ward patients. J Patient Saf.

[44] McNeill G, Bryden D (2013). Do either early warning systems or emergency response teams improve hospital patient survival? A systematic review. Resuscitation.

[45] Stolze A, Woolley-Hendriks TNM, Bassa Y (2024). The effect of early warning scoring systems on adverse outcome in surgical patients: a systematic review. Int J Nurs Stud Adv.

[46] Smith MEB, Chiovaro JC, O’Neil M (2014). Early warning system scores for clinical deterioration in hospitalized patients: a systematic review. Ann Am Thorac Soc.

[47] Nydahl P, Jeitziner MM, Krotsetis S Early warning scores: a rapid umbrella review. Med Klin Intensivmed Notfmed.

[48] Edelson DP, Churpek MM, Carey KA (2024). Early warning scores with and without artificial intelligence. JAMA Netw Open.

[49] Hincapié-Osorno C, van Wijk RJ, Postma DF (2024). Validation of MEWS, NEWS, NEWS-2 and qSOFA for different infection foci at the emergency department, the acutelines cohort. Eur J Clin Microbiol Infect Dis.

