# Supplementary material for: Efficiency of Early Warning Scores in Adult Emergency Department and Inpatient Populations: Overview of Reviews
Source: West J Emerg Med. 2026 Aug 31;27(5):1222–34. doi: 10.5811/westjem.62022 (PMC13660599; doi:10.5811/westjem.62022)
Supplement: Supplementary file 1 [file wjem-27-1222-s001.docx]

**Ovid MEDLINE(R) ALL 1946 to May 01, 2025**

**The search was conducted on 2nd May 2025 at 10:00 am (CET).**

| **#** | **search string** | **# of results** |
| --- | --- | --- |
| 1 | Early Warning Score/ | 531 |
| 2 | (early warning adj3 scor*).tw,kf,kw. | 2347 |
| 3 | early warning system?.kf,kw,tw. | 3566 |
| 4 | Physiological social scor*.kf,kw,tw. | 5 |
| 5 | or/1-4 | 5752 |
| 6 | (systematic review or meta-analysis).pt. | 378609 |
| 7 | meta-analysis/ or systematic review/ or systematic reviews as topic/ or meta-analysis as topic/ or "meta analysis (topic)"/ or "systematic review (topic)"/ or exp technology assessment, biomedical/ or network meta-analysis/ | 424099 |
| 8 | ((systematic* adj3 (review* or overview*)) or (methodologic* adj3 (review* or overview*))).ti,ab,kf. | 415512 |
| 9 | ((quantitative adj3 (review* or overview* or synthes*)) or (research adj3 (integrati* or overview*))).ti,ab,kf. | 19790 |
| 10 | ((integrative adj3 (review* or overview*)) or (collaborative adj3 (review* or overview*)) or (pool* adj3 analy*)).ti,ab,kf. | 46629 |
| 11 | (data synthes* or data extraction* or data abstraction*).ti,ab,kf. | 51431 |
| 12 | (handsearch* or hand search*).ti,ab,kf. | 12029 |
| 13 | (mantel haenszel or peto or der simonian or dersimonian or fixed effect* or latin square*).ti,ab,kf. | 41418 |
| 14 | (met analy* or metanaly* or technology assessment* or HTA or HTAs or technology overview* or technology appraisal*).ti,ab,kf. | 14142 |
| 15 | (meta regression* or metaregression*).ti,ab,kf. | 18502 |
| 16 | (meta-analy* or metaanaly* or systematic review* or biomedical technology assessment* or bio-medical technology assessment*).mp,hw. | 574255 |
| 17 | (medline or cochrane or pubmed or medlars or embase or cinahl).ti,ab,hw. | 429413 |
| 18 | (cochrane or (health adj2 technology assessment) or evidence report).jw. | 22511 |
| 19 | (comparative adj3 (efficacy or effectiveness)).ti,ab,kf. | 21215 |
| 20 | (outcomes research or relative effectiveness).ti,ab,kf. | 12350 |
| 21 | ((indirect or indirect treatment or mixed-treatment or bayesian) adj3 comparison*).ti,ab,kf. | 4994 |
| 22 | (multi* adj3 treatment adj3 comparison*).ti,ab,kf. | 332 |
| 23 | (mixed adj3 treatment adj3 (meta-analy* or metaanaly*)).ti,ab,kf. | 185 |
| 24 | umbrella review*.ti,ab,kf. | 2744 |
| 25 | (multi* adj2 paramet* adj2 evidence adj2 synthesis).ti,ab,kf. | 15 |
| 26 | (multiparamet* adj2 evidence adj2 synthesis).ti,ab,kf. | 19 |
| 27 | (multi-paramet* adj2 evidence adj2 synthesis).ti,ab,kf. | 13 |
| 28 | or/6-27 | 835199 |
| 29 | 5 and 28 | 298 |

**EPISTEMONIKOS**

**The search was conducted on 2nd May 2025 at 10:15 am (CET).**

| **#** | **search string** | **# of results** |
| --- | --- | --- |
| **1** | (title:("early warning score" OR "early warning scoring" OR "early warning scores" OR "early warning system" OR "early warning systems" OR "Physiological social scoring" OR "Physiological social scores" OR "Physiological social score" OR "early warning signs scoring" OR "early warning signs score" OR "early warning signs scores") OR abstract:("early warning score" OR "early warning scoring" OR "early warning scores" OR "early warning system" OR "early warning systems" OR "Physiological social scoring" OR "Physiological social scores" OR "Physiological social score" OR "early warning signs scoring" OR "early warning signs score" OR "early warning signs scores")) | 2167 |
| **2** | Broad synthesis | 16 |
| **3** | Structured summary | 11 |
| **4** | Systematic review | 177 |
