# Supplementary material for: Efficiency of Early Warning Scores in Adult Emergency Department and Inpatient Populations: Overview of Reviews
Source: West J Emerg Med. 2026 Aug 31;27(5):1222–34. doi: 10.5811/westjem.62022 (PMC13660599; doi:10.5811/westjem.62022)
Supplement: Supplementary file 2 [file wjem-27-1222-s003.docx]

List of excluded studies

| 1 | Baig MM, GholamHosseini H, Afifi S, Lindén M. A systematic review of rapid response applications based on early warning score for early detection of inpatient deterioration. Inform Health Soc Care. 2021 Jun 2;46(2):148-157. doi: 10.1080/17538157.2021.1873349. Epub 2021 Jan 21. PMID: 33472485. | Not eligible intervention |
| --- | --- | --- |
| 2 | Bernstein S. Scoping Review on the Use of Early Warning Trigger Tools for Women in Labor. J Obstet Gynecol Neonatal Nurs. 2021 May;50(3):256-265. doi: 10.1016/j.jogn.2021.01.003. Epub 2021 Feb 5. PMID: 33549533. | Not eligible population (obstetric), not eligible study design (scoping review) |
| 3 | Blythe R, Parsons R, White NM, Cook D, McPhail S. A scoping review of real-time automated clinical deterioration alerts and evidence of impacts on hospitalised patient outcomes. BMJ Qual Saf. 2022 Oct;31(10):725-734. doi: 10.1136/bmjqs-2021-014527. Epub 2022 Jun 22. PMID: 35732487. | Not eligible study design (scoping review) |
| 4 | Brekke IJ, Puntervoll LH, Pedersen PB, Kellett J, Brabrand M. The value of vital sign trends in predicting and monitoring clinical deterioration: A systematic review. PLoS One. 2019 Jan 15;14(1):e0210875. doi: 10.1371/journal.pone.0210875. PMID: 30645637; PMCID: PMC6333367. | Not eligible intervention |
| 5 | Burgos-Esteban A, Gea-Caballero V, Marín-Maicas P, Santillán-García A, Cordón-Hurtado MV, Marqués-Sule E, Giménez-Luzuriaga M, Juárez-Vela R, Sanchez-Gonzalez JL, García-Criado J, Santolalla-Arnedo I. Effectiveness of Early Warning Scores for Early Severity Assessment in Outpatient Emergency Care: A Systematic Review. Front Public Health. 2022 Jul 14;10:894906. doi: 10.3389/fpubh.2022.894906. PMID: 35910902; PMCID: PMC9330632. | Not eligible population (pre-hospital) |
| 6 | Burke C, Conway Y. Factors that influence hospital nurses' escalation of patient care in response to their early warning score: A qualitative evidence synthesis. J Clin Nurs. 2023 May;32(9-10):1885-1934. doi: 10.1111/jocn.16233. Epub 2022 Mar 25. PMID: 35338540. | Not eligible intervention |
| 7 | Chua, Wei Ling et al. “Early warning scores for sepsis identification and prediction of in-hospital mortality in adults with sepsis: A systematic review and meta-analysis.” Journal of clinical nursing vol. 33,6 (2024): 2005-2018. doi:10.1111/jocn.17061 | Not eligible population (incl. pre-hospital) |
| 8 | Chua WL, See MTA, Legio-Quigley H, Jones D, Tee A, Liaw SY. Factors influencing the activation of the rapid response system for clinically deteriorating patients by frontline ward clinicians: a systematic review. Int J Qual Health Care. 2017 Dec 1;29(8):981-998. doi: 10.1093/intqhc/mzx149. Erratum in: Int J Qual Health Care. 2018 Jul 1;30(6):492. doi: 10.1093/intqhc/mzy040. PMID: 29177454; PMCID: PMC6216047. | Not eligible intervention |
| 9 | Connolly F, Byrne D, Lydon S, Walsh C, O'Connor P. Barriers and facilitators related to the implementation of a physiological track and trigger system: A systematic review of the qualitative evidence. Int J Qual Health Care. 2017 Dec 1;29(8):973-980. doi: 10.1093/intqhc/mzx148. PMID: 29177409. | Not eligible intervention |
| 10 | Credland N, Dyson J, Johnson MJ. What are the patterns of compliance with Early Warning Track and Trigger Tools: A narrative review. Appl Nurs Res. 2018 Dec;44:39-47. doi: 10.1016/j.apnr.2018.09.002. Epub 2018 Sep 18. PMID: 30389058. | Not eligible intervention |
| 11 | Dall'Ora C, Griffiths P, Hope J, Barker H, Smith GB. What is the nursing time and workload involved in taking and recording patients' vital signs? A systematic review. J Clin Nurs. 2020 Jul;29(13-14):2053-2068. doi: 10.1111/jocn.15202. Epub 2020 Feb 3. PMID: 32017272. | Not eligible intervention |
| 12 | Downey CL, Tahir W, Randell R, Brown JM, Jayne DG. Strengths and limitations of early warning scores: A systematic review and narrative synthesis. Int J Nurs Stud. 2017 Nov;76:106-119. doi: 10.1016/j.ijnurstu.2017.09.003. Epub 2017 Sep 13. PMID: 28950188. | Not eligible population (pre-hospital) |
| 13 | Ede J, Petrinic T, Westgate V, Darbyshire J, Endacott R, Watkinson PJ. Human factors in escalating acute ward care: a qualitative evidence synthesis. BMJ Open Qual. 2021 Feb;10(1):e001145. doi: 10.1136/bmjoq-2020-001145. PMID: 33637554; PMCID: PMC7919590. | Not eligible intervention |
| 14 | Esmaeilzadeh S, Lane CM, Gerberi DJ, Wakeam E, Pickering BW, Herasevich V, Hyder JA. Improving In-Hospital Patient Rescue: What Are Studies on Early Warning Scores Missing? A Scoping Review. Crit Care Explor. 2022 Feb 21;4(2):e0644. doi: 10.1097/CCE.0000000000000644. PMID: 35224506; PMCID: PMC8863168. | Not eligible outcome, not eligible study design (scoping review) |
| 15 | Fang AHS, Lim WT, Balakrishnan T. Early warning score validation methodologies and performance metrics: a systematic review. BMC Med Inform Decis Mak. 2020 Jun 18;20(1):111. doi: 10.1186/s12911-020-01144-8. PMID: 32552702; PMCID: PMC7301346. | Not eligible outcome |
| 16 | Fang AHS, Lim WT, Balakrishnan T. Early warning score validation methodologies and performance metrics: a systematic review. BMC Med Inform Decis Mak. 2020 Jun 18;20(1):111. doi: 10.1186/s12911-020-01144-8. PMID: 32552702; PMCID: PMC7301346. | Not eligible intervention |
| 17 | Fu LH, Schwartz J, Moy A, Knaplund C, Kang MJ, Schnock KO, Garcia JP, Jia H, Dykes PC, Cato K, Albers D, Rossetti SC. Development and validation of early warning score system: A systematic literature review. J Biomed Inform. 2020 May;105:103410. doi: 10.1016/j.jbi.2020.103410. Epub 2020 Apr 8. PMID: 32278089; PMCID: PMC7295317. | Not eligible intervention |
| 18 | Gerry S, Bonnici T, Birks J, Kirtley S, Virdee PS, Watkinson PJ, Collins GS. Early warning scores for detecting deterioration in adult hospital patients: systematic review and critical appraisal of methodology. BMJ. 2020 May 20;369:m1501. doi: 10.1136/bmj.m1501. PMID: 32434791; PMCID: PMC7238890. | Not eligible outcome |
| 19 | Guan G, Lee CMY, Begg S, Crombie A, Mnatzaganian G. The use of early warning system scores in prehospital and emergency department settings to predict clinical deterioration: A systematic review and meta-analysis. PLoS One. 2022 Mar 17;17(3):e0265559. doi: 10.1371/journal.pone.0265559. PMID: 35298560; PMCID: PMC8929648. | Not eligible population (prehospital) |
| 20 | Halverson CC, Bailey C, Ennis JA, Cox EE. Nursing surveillance of respiratory adverse events among hospitalized adults: A systematic review to guide evidence-based practice. Worldviews Evid Based Nurs. 2022 Aug;19(4):260-266. doi: 10.1111/wvn.12581. Epub 2022 May 31. PMID: 35638706. | Not eligible intervention |
| 21 | Hogan H, Hutchings A, Wulff J, Carver C, Holdsworth E, Welch J, Harrison D, Black N. Interventions to reduce mortality from in-hospital cardiac arrest: a mixed-methods study. Southampton (UK): NIHR Journals Library; 2019 Jan. PMID: 30676710. | Not eligible study design |
| 22 | Holland M, Kellett J. A systematic review of the discrimination and absolute mortality predicted by the National Early Warning Scores according to different cut-off values and prediction windows. Eur J Intern Med. 2022 Apr;98:15-26. doi: 10.1016/j.ejim.2021.12.024. Epub 2021 Dec 31. PMID: 34980504. | Not eligible population (pre-hospital) |
| 23 | Jensen JK, Skår R, Tveit B. The impact of Early Warning Score and Rapid Response Systems on nurses' competence: An integrative literature review and synthesis. J Clin Nurs. 2018 Apr;27(7-8):e1256-e1274. doi: 10.1111/jocn.14239. Epub 2018 Feb 8. PMID: 29274170. | Not eligible population |
| 24 | Khalifa M, Magrabi F, Gallego B. Developing a framework for evidence-based grading and assessment of predictive tools for clinical decision support. BMC Med Inform Decis Mak. 2019 Oct 29;19(1):207. doi: 10.1186/s12911-019-0940-7. PMID: 31664998; PMCID: PMC6820933. | Not eligible intervention |
| 25 | Kumar A, Abbenbroek B, Delaney A, Hammond N, Grattan S, Finfer S. Sepsis triggers and tools to support early identification in healthcare settings: An integrative review. Aust Crit Care. 2023 Nov;36(6):1117-1128. doi: 10.1016/j.aucc.2023.01.001. Epub 2023 Feb 20. PMID: 36813654. | Not eligible population |
| 26 | Le Lagadec MD, Dwyer T. Scoping review: The use of early warning systems for the identification of in-hospital patients at risk of deterioration. Aust Crit Care. 2017 Jul;30(4):211-218. doi: 10.1016/j.aucc.2016.10.003. Epub 2016 Nov 15. PMID: 27863876. | Not eligible study design (scoping review) |
| 27 | Linnen DT, Escobar GJ, Hu X, Scruth E, Liu V, Stephens C. Statistical Modeling and Aggregate-Weighted Scoring Systems in Prediction of Mortality and ICU Transfer: A Systematic Review. J Hosp Med. 2019 Mar;14(3):161-169. doi: 10.12788/jhm.3151. PMID: 30811322; PMCID: PMC6628701. | Not eligible intervention |
| 28 | Maciver M. Pre-hospital use of early warning scores to improve detection and outcomes of sepsis. Br J Community Nurs. 2021 Mar 2;26(3):122-129. doi: 10.12968/bjcn.2021.26.3.122. PMID: 33719557. | Not eligible population (pre-hospital) |
| 29 | Mapp ID, Davis LL, Krowchuk H. Prevention of unplanned intensive care unit admissions and hospital mortality by early warning systems. Dimens Crit Care Nurs. 2013 Nov-Dec;32(6):300-9. doi: 10.1097/DCC.0000000000000004. PMID: 24100432. | Not eligible study design (high risk of bias) |
| 30 | McArthur-Rouse F. Critical care outreach services and early warning scoring systems: a review of the literature. J Adv Nurs. 2001 Dec;36(5):696-704. doi: 10.1046/j.1365-2648.2001.02020.x. PMID: 11737502. | Not eligible outcome |
| 31 | McGaughey J, Alderdice F, Fowler R, Kapila A, Mayhew A, Moutray M. Outreach and Early Warning Systems (EWS) for the prevention of intensive care admission and death of critically ill adult patients on general hospital wards. Cochrane Database Syst Rev. 2007 Jul 18;(3):CD005529. doi: 10.1002/14651858.CD005529.pub2. Update in: Cochrane Database Syst Rev. 2021 Nov 22;11:CD005529. doi: 10.1002/14651858.CD005529.pub3. PMID: 17636805. | Not eligible study design (The old version of the updated Cochrane review) |
| 32 | McGaughey J, O'Halloran P, Porter S, Blackwood B. Early warning systems and rapid response to the deteriorating patient in hospital: A systematic realist review. J Adv Nurs. 2017 Dec;73(12):2877-2891. doi: 10.1111/jan.13398. Epub 2017 Sep 20. PMID: 28727184. | Not eligible intervention |
| 33 | Smith MEB, Chiovaro JC, O'Neil M, Kansagara D, Quinones A, Freeman M, Motu'apuaka M, Slatore CG. Early Warning System Scores: A Systematic Review [Internet]. Washington (DC): Department of Veterans Affairs (US); 2014 Jan. PMID: 25506953. | The research is published in two articles |
| 34 | McNeill H, Khairat S. Impact of Intensive Care Unit Readmissions on Patient Outcomes and the Evaluation of the National Early Warning Score to Prevent Readmissions: Literature Review. JMIR Perioper Med. 2020 May 8;3(1):e13782. doi: 10.2196/13782. PMID: 33393911; PMCID: PMC7709858. | Not eligible intervention |
| 35 | Moffat LM, Xu D. Accuracy of Machine Learning Models to Predict In-hospital Cardiac Arrest: A Systematic Review. Clin Nurse Spec. 2022 Jan-Feb 01;36(1):29-44. doi: 10.1097/NUR.0000000000000644. PMID: 34843192. | Not eligible intervention |
| 36 | Muralitharan S, Nelson W, Di S, McGillion M, Devereaux PJ, Barr NG, Petch J. Machine Learning-Based Early Warning Systems for Clinical Deterioration: Systematic Scoping Review. J Med Internet Res. 2021 Feb 4;23(2):e25187. doi: 10.2196/25187. PMID: 33538696; PMCID: PMC7892287. | Not eligible study design (scoping review) |
| 37 | Murphy A, Cronin J, Whelan R, Drummond FJ, Savage E, Hegarty J. Economics of Early Warning Scores for identifying clinical deterioration-a systematic review. Ir J Med Sci. 2018 Feb;187(1):193-205. doi: 10.1007/s11845-017-1631-y. Epub 2017 Jun 3. PMID: 28580508. | Not eligible study design |
| 38 | Oanesa RD, Su TW, Weissman A. Evidence for Use of Validated Sepsis Screening Tools in the Prehospital Population: A Scoping Review. Prehosp Emerg Care. 2024;28(3):485-493. doi: 10.1080/10903127.2023.2224862. Epub 2023 Jul 6. PMID: 37327065. | Not eligible population (pre-hospital) |
| 39 | Odell M. Are early warning scores the only way to rapidly detect and manage deterioration? Nurs Times. 2010 Mar 2-8;106(8):24-6. PMID: 20334002. | Not eligible study design (literature review) |
| 40 | O'Neill SM, Clyne B, Bell M, Casey A, Leen B, Smith SM, Ryan M, O'Neill M. Why do healthcare professionals fail to escalate as per the early warning system (EWS) protocol? A qualitative evidence synthesis of the barriers and facilitators of escalation. BMC Emerg Med. 2021 Jan 28;21(1):15. doi: 10.1186/s12873-021-00403-9. PMID: 33509099; PMCID: PMC7842002. | Not eligible intervention |
| 41 | Patel R, Nugawela MD, Edwards HB, Richards A, Le Roux H, Pullyblank A, Whiting P. Can early warning scores identify deteriorating patients in pre-hospital settings? A systematic review. Resuscitation. 2018 Nov;132:101-111. doi: 10.1016/j.resuscitation.2018.08.028. Epub 2018 Aug 29. PMID: 30171976. | Not eligible population (pre-hospital) |
| 42 | Roney JK, Whitley BE, Maples JC, Futrell LS, Stunkard KA, Long JD. Modified early warning scoring (MEWS): evaluating the evidence for tool inclusion of sepsis screening criteria and impact on mortality and failure to rescue. J Clin Nurs. 2015 Dec;24(23-24):3343-54. doi: 10.1111/jocn.12952. Epub 2015 Aug 12. PMID: 26780181. | Not eligible study design |
| 43 | Saab MM, McCarthy B, Andrews T, Savage E, Drummond FJ, Walshe N, Forde M, Breen D, Henn P, Drennan J, Hegarty J. The effect of adult Early Warning Systems education on nurses' knowledge, confidence and clinical performance: A systematic review. J Adv Nurs. 2017 Nov;73(11):2506-2521. doi: 10.1111/jan.13322. Epub 2017 May 22. PMID: 28440892. | Not eligible population; not eligible outcome |
| 44 | Smith V, Kenny LC, Sandall J, Devane D, Noonan M. Physiological track-and-trigger/early warning systems for use in maternity care. Cochrane Database Syst Rev. 2021 Sep 13;9(9):CD013276. doi: 10.1002/14651858.CD013276.pub2. PMID: 34515991; PMCID: PMC8436732. | Not eligible population (obstetric) |
| 45 | Smith V, O'Malley D, Cithambaram K. Early warning systems in maternity care: A qualitative evidence synthesis of maternity care providers' views and experiences. Midwifery. 2022 Sep;112:103402. doi: 10.1016/j.midw.2022.103402. Epub 2022 Jun 11. PMID: 35724435. | Not eligible population (obstetric) |
| 46 | Subbe CP, Williams E, Fligelstone L, Gemmell L. Does earlier detection of critically ill patients on surgical wards lead to better outcomes? Ann R Coll Surg Engl. 2005 Jul;87(4):226-32. doi: 10.1308/003588405X50921. PMID: 16053678; PMCID: PMC1963939. | Not eligible study design |
| 47 | Suwanpasu S. & Sattayasomboon Y. (2016). Modified early warning score to predict mortality in hospital: A systematic review and meta-analysis. Intensive Care Medicine Experimental. EPISTEMONIKOS. https://doi.org/10.1186/s40635-016-0099-9 | Not eligible study design (conference abstract) |
| 48 | Treacy M, Caroline Stayt L. To identify the factors that influence the recognizing and responding to adult patient deterioration in acute hospitals. J Adv Nurs. 2019 Dec;75(12):3272-3285. doi: 10.1111/jan.14138. Epub 2019 Jul 30. PMID: 31241199. | No eligible intervention |
| 49 | Umar A, Ameh CA, Muriithi F, Mathai M. Early warning systems in obstetrics: A systematic literature review. PLoS One. 2019 May 31;14(5):e0217864. doi: 10.1371/journal.pone.0217864. PMID: 31150513; PMCID: PMC6544303. | Not eligible population (obstetric) |
| 50 | Veldhuis LI, Ridderikhof ML, Bergsma L, Van Etten-Jamaludin F, Nanayakkara PW, Hollmann M. Performance of early warning and risk stratification scores versus clinical judgement in the acute setting: a systematic review. Emerg Med J. 2022 Dec;39(12):918-923. doi: 10.1136/emermed-2021-211524. Epub 2022 Aug 9. PMID: 35944968. | Not eligible population (pre-hospital) |
| 51 | Wei S, Xiong D, Wang J, Liang X, Wang J, Chen Y. The accuracy of the National Early Warning Score 2 in predicting early death in prehospital and emergency department settings: a systematic review and meta-analysis. Ann Transl Med. 2023 Jan 31;11(2):95. doi: 10.21037/atm-22-6587. PMID: 36819553; PMCID: PMC9929743. | Not eligible population (pre-hospital) |
| 52 | Williams TA, Tohira H, Finn J, Perkins GD, Ho KM. The ability of early warning scores (EWS) to detect critical illness in the prehospital setting: A systematic review. Resuscitation. 2016 May;102:35-43. doi: 10.1016/j.resuscitation.2016.02.011. Epub 2016 Feb 22. PMID: 26905389. | Not eligible population (pre-hospital) |
| 53 | Wood C, Chaboyer W, Carr P. How do nurses use early warning scoring systems to detect and act on patient deterioration to ensure patient safety? A scoping review. Int J Nurs Stud. 2019 Jun;94:166-178. doi: 10.1016/j.ijnurstu.2019.03.012. Epub 2019 Mar 23. PMID: 31002971. | Not eligible study design (scoping review) |
| 54 | Zhang H, Jiang JX, Xia J, Yu C, Zhong MH, Pang QY, Mao YL, Duan X. Analysis of clinical application effects and challenges of early warning system for the high-risk obstetric women of China: A scoping review. J Clin Nurs. 2023 May;32(9-10):2073-2085. doi: 10.1111/jocn.16288. Epub 2022 Mar 18. PMID: 35304785. | Not eligible population (obstetric) |
