# Supplementary material for: Efficiency of Early Warning Scores in Adult Emergency Department and Inpatient Populations: Overview of Reviews
Source: West J Emerg Med. 2026 Aug 31;27(5):1222–34. doi: 10.5811/westjem.62022 (PMC13660599; doi:10.5811/westjem.62022)
Supplement: Supplementary file 3 [file wjem-27-1222-s004.docx]

Graphical Representation of Overlap for OVErviews (GROOVE)

|  | Adegbite |  |  |  |  |  |  |  |  |  |  |  |  |  |  |  |  |  |  |  |
| --- | --- | --- | --- | --- | --- | --- | --- | --- | --- | --- | --- | --- | --- | --- | --- | --- | --- | --- | --- | --- |
|  |  | Alam | Alhmoud Baneen | Arévalo-Buitrago | Credland Nicola | Hamilton F | Jayasundera Romesh | Ju-Ry Lee | Long Jianying |  |  |  |  |  |  |  |  |  |  |  |
|  |  |  |  |  |  |  |  |  |  | **210** | **=** | **Total nodes (pairs of reviews)** | | | | |  |  |  |  |
| Alam | 0,0% |  |  |  |  |  |  |  |  | 176 | = | Slight overlap (<5%) | | | | |  |  |  |  |
| Alhmoud Baneen | 3,3% | 0,0% |  |  |  |  |  |  |  | 18 | = | Moderate overlap (5% to <10%) | | | | |  |  |  |  |
| Arévalo-Buitrago | 0,0% | 6,7% | 2,8% |  |  |  |  |  |  | 9 | = | High overlap (10% to <15%) | | | | |  |  |  |  |
| Credland Nicola | 0,0% | 0,0% | 0,9% | 7,7% |  |  |  |  |  | 7 | = | Very High overlap (≥15%) | | | | |  |  |  |  |
| Hamilton F | 0,0% | 0,0% | 4,8% | 0,0% | 0,0% |  |  |  |  | McGaughey J | McNeill | Nannan | Qiu Xia | Sabir Lisa | Smith a (deterioration in hosp.) | Wang | Wuytack | Zhang a/National Early Warning Score Does | Zhang b/The Prognostic Accurac | Lin Lan |
| Jayasundera Romesh | 0,0% | 0,0% | 0,9% | 0,0% | 0,0% | 0,0% |  |  |  |  |  |  |  |  |  |  |  |  |  |  |
| Ju-Ry Lee | 0,0% | 8,3% | 0,0% | 7,1% | 10,0% | 0,0% | 0,0% |  |  |  |  |  |  |  |  |  |  |  |  |  |
| Long Jianying | 0,0% | 0,0% | 0,0% | 0,0% | 0,0% | 0,0% | 0,0% | 3,0% |  |  |  |  |  |  |  |  |  |  |  |  |
| McGaughey J | 0,0% | 0,0% | 0,9% | 0,0% | 0,0% | 0,0% | 0,0% | 0,0% | 0,0% |  |  |  |  |  |  |  |  |  |  |  |
| McNeill | 0,0% | 22,2% | 0,0% | 0,0% | 0,0% | 0,0% | 0,0% | 11,1% | 0,0% | 0,0% |  |  |  |  |  |  |  |  |  |  |
| Nannan | 0,0% | 2,1% | 16,0% | 10,9% | 4,4% | 9,1% | 2,2% | 2,1% | 0,0% | 0,0% | 0,0% |  |  |  |  |  |  |  |  |  |
| Qiu Xia | 6,6% | 0,0% | 3,2% | 0,0% | 0,0% | 0,0% | 0,0% | 0,0% | 0,0% | 0,0% | 0,0% | 1,0% |  |  |  |  |  |  |  |  |
| Sabir Lisa | 0,0% | 0,0% | 4,5% | 0,0% | 0,0% | 0,0% | 0,0% | 0,0% | 0,0% | 0,0% | 0,0% | 1,9% | 6,1% |  |  |  |  |  |  |  |
| Smith a (deterioration in hosp.) | 0,0% | 16,7% | 2,5% | 0,0% | 4,0% | 0,0% | 0,0% | 3,8% | 0,0% | 0,0% | 13,6% | 3,3% | 0,0% | 3,0% |  |  |  |  |  |  |
| Wang | 4,2% | 0,0% | 4,0% | 0,0% | 0,0% | 0,0% | 0,0% | 0,0% | 0,0% | 0,0% | 0,0% | 0,0% | 12,2% | 8,3% | 0,0% |  |  |  |  |  |
| Wuytack | 0,0% | 0,0% | 11,2% | 7,1% | 2,5% | 5,0% | 0,0% | 0,0% | 0,0% | 0,0% | 0,0% | 25,8% | 0,0% | 0,0% | 0,0% | 1,6% |  |  |  |  |
| Zhang a/National Early Warning Score Does | 4,7% | 0,0% | 7,8% | 0,0% | 0,0% | 3,8% | 0,0% | 0,0% | 0,0% | 0,0% | 0,0% | 1,6% | 11,4% | 21,4% | 0,0% | 23,7% | 1,8% |  |  |  |
| Zhang b/The Prognostic Accurac | 0,0% | 0,0% | 0,8% | 0,0% | 0,0% | 0,0% | 0,0% | 0,0% | 0,0% | 0,0% | 0,0% | 1,6% | 1,3% | 0,0% | 0,0% | 0,0% | 1,8% | 0,0% |  |  |
| Lin Lan | 3,8% | 0,0% | 3,1% | 0,0% | 0,0% | 2,9% | 0,0% | 0,0% | 0,0% | 0,0% | 0,0% | 1,4% | 8,8% | 7,5% | 0,0% | 12,0% | 0,0% | 10,9% | 0,0% |  |
| Stolze | 0,0% | 7,1% | 0,0% | 0,0% | 8,3% | 0,0% | 0,0% | 16,7% | 0,0% | 5,6% | 9,1% | 0,0% | 0,0% | 0,0% | 7,4% | 0,0% | 0,0% | 0,0% | 0,0% | 0,0% |
